# Cardiac Mapping: Utility or Futility?

**Published:** 2002-01-01

**Authors:** Anoop Kumar Gupta, Alok Maheshwari, Ranjan Thakur, Yash Y Lokhandwala

**Affiliations:** Thoracic and Cardiovascular Institute, Sparrow Health System, Michigan State University, Lansing, MI. USA

## Introduction

Cardiac mapping is a broad term that covers several modes of mapping such as body surface [1], endocardial [2], and epicardial [3] mapping. The recording and analysis of extracellular electrograms, reported as early as 1915, forms the basis for cardiac mapping [4]. More commonly, cardiac mapping is performed with catheters that are introduced percutaneously into the heart chambers and sequentially record the endocardial electrograms with the purpose of correlating local electrogram to cardiac anatomy. These electrophysiological catheters are navigated and localized with the use of fluoroscopy. Nevertheless, the use of fluoroscopy for these purposes may be problematic for a number of reasons, including: 1) the inability to accurately associate intracardiac electrograms with their precise location within the heart; 2) the endocardial surface is invisible using fluoroscopy and the target sites can only be approximated by their relationship with nearby structures such as ribs, blood vessels, and the position of other catheters; 3) due to the limitations of two-dimensional fluoroscopy, navigation is not exact, time consuming, and requires multiple views to estimate the three-dimensional location of the catheter; 4) inability to accurately return the catheter precisely to a previously mapped site; and 5) exposure of the patient and medical team to radiation.

Newer mapping systems have revolutionized the clinical electrophysiology laboratory in recent years and have offered new insights into arrhythmia mechanisms. They are aimed at improving the resolution, three-dimensional spatial localization, and/or rapidity of acquisition of cardiac activation maps. These systems use novel approaches to accurately determine the three-dimensional location of the mapping catheter and local electrograms are acquired using conventional, well-established methods. Recorded data of the catheter location and intracardiac electrogram at that location are used to reconstruct in real-time a representation of the three-dimensional geometry of the chamber, color-coded with relevant electrophysiological information.

However, these mapping systems are very expensive and not required for the commoner clinical arrhythmias like atrioventricular nodal reentry (AVNRT), accessory pathway mediated tachycardia (WPW syndrome and concealed pathways) and typical atrial flutter. The purpose of this article is to discuss the possible contribution of newer cardiac mapping system to treat various arrhythmias.

## Non-contact Endocardial mapping

### Endocardial Solutions (ESI) system

Myocardial potentials are reconstructed from data acquired by recording cavity potential from an array of electrodes sitting in the blood pool within the cardiac chamber and not in endocardial contact. Inverse- solution methods are applied to these non-contact signals in order to reconstruct endocardial potentials from the raw cavity potentials.

**Figure 1:**
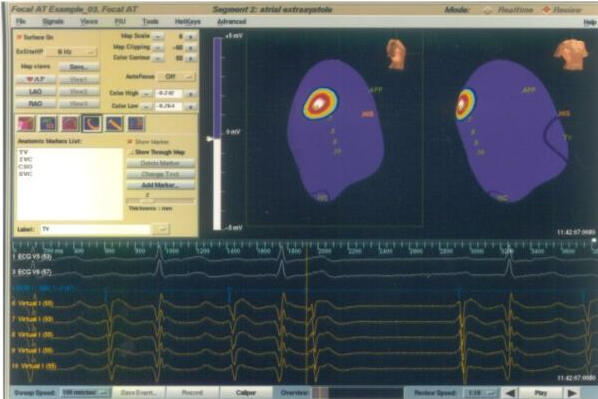
Endocardial Solutions mapping showing the earliest activation (in red color) in a case of focal atrial tachycardia. The upper right hand panel displays AP projection on the left and LAO projection on the right side. The lower half of the picture shows intracardiac tracings. TV= Tricuspid annulus; IVC= Inferior vena cava; HIS= His bundle; APP= Anteroposterior projection.

**Figure 2:**
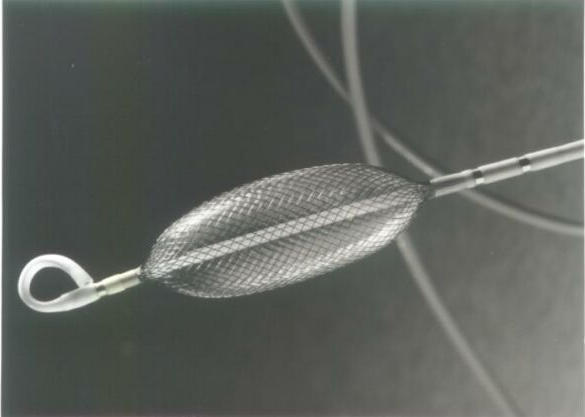
The Endocardial Solutions electrode array mounted on a 9F catheter.

Endocardial Solutions (ESI) system is used for clinical non-contact intracardiac mapping. It can display high-resolution color maps of endocardial activation in the intact beating heart (Figure 1). The electrode array is mounted on a 9F catheter and consists of a 7.5 ml balloon around which is woven a braid of 64 insulated 0.003 inch diameter wires, with a single break in its insulation, producing 64 non-contact unipolar electrodes (Figure 2). A multi-channel amplifier and computer workstation processes the raw far-field electrographic data. The system is applied in three steps:

1. Cardiac chamber geometry is established.
2. Site(s) critical for maintenance of reentry circuit(s) are identified.
3. Ablation catheter is navigated to critical site(s).

A catheter locator system is central to steps 1 and 3 while the inverse solution for reconstructing endocardial electrograms is central to step 2. A 'locator' signal emitted from the mapping/ablation catheter allows its position to be determined relative to the electrode array. It can be used to construct the three-dimensional computer model of the endocardium (virtual endocardium) by moving the conventional catheter around the cardiac chamber, thus building a series of co-ordinates for the endocardium. Reconstructed electrograms are then superimposed onto the virtual endocardium to produce isopotential maps.

Electrogram reconstruction has been validated by comparing the morphology and timing of reconstructed electrograms with contact electrograms from the same endocardial location (as indicated by the catheter location system). The accuracy of electrogram reconstruction is good, but decreases with increasing distance between the electrode array and the endocardium and this becomes significant for distances >34 mm.

The main advantage of this system is that it requires only one beat to reconstruct a complete activation map, which potentially allows mapping of hemodynamically unstable arrhythmias. However, the distance between the endocardial wall and the balloon center and the spatial complexity of the activation patterns influences the accuracy of the electrogram reconstruction negatively. This will affect the reliability when mapping for example, dilated LVs or complex reentrant circuits [5-7].

## Contact Mapping system

### 1) Electroanatomical mapping (CARTO System)

The technology is based on a catheter location system, which determines a mapping catheter's position and attitude within an ultra low magnetic field emitted from radiators positioned under the operating table.

#### System Components

The catheter resembles a standard 7- or 8- Fr deflectable catheter with a 4-mm tip and proximal 2-mm ring electrodes. The location sensors lie just proximal to the tip electrode, totally embedded within the catheter. The three location sensors are located orthogonally to each other. The locator pad is placed beneath the operating table and includes three coils that generate ultralow magnetic fields, which decay as a function of the distance from their sources. When the catheter is moved within this magnetic field, the currents generated represent movement in three dimensions. The resolution of the location capabilities of the system has been previously been shown to be <1 mm in both in vitro and in vivo studies [8]. This information enables tracking of the tip of the mapping catheter within the heart, enabling navigation of the catheter independent of fluoroscopy. Signals received within the sensor are transmitted along the catheter shaft to the main processing unit.

#### Mapping procedure

The reference catheter is introduced and placed inside the coronary sinus or in the right ventricle. The mapping/ ablation catheter is introduced and placed in the chamber being mapping. The mapping system determines the location and orientation of both the mapping and reference catheters. The location of the mapping catheter is gated to a fiducial point in the cardiac cycle and recorded relative to the location of the fixed reference catheter at that time, thus compensated for both subject and cardiac motion. By moving the catheter inside the heart, the system continuously analyzes its location and presents it to the user, thus enabling navigation without the use of fluoroscopy.

**Figure 3:**
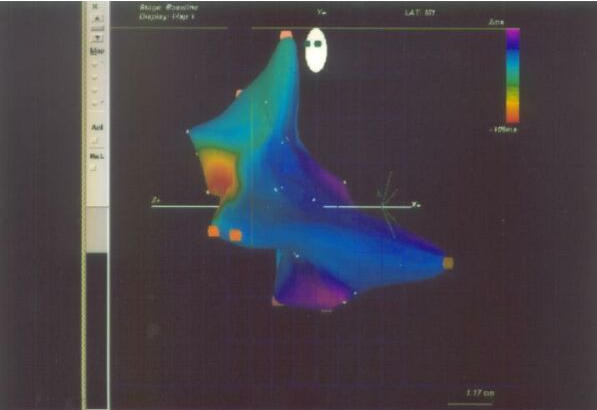

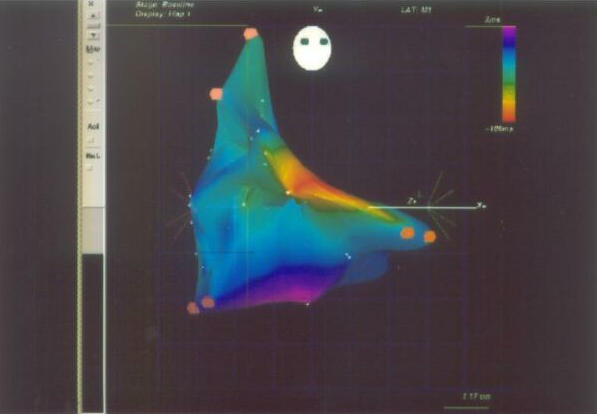
Electroanatomic mapping on CARTO system of focal right atrial tachycardia showing  earliest activation (red color) below crista terminalis. LAO 40° projection ( Figure 3A) and RAO 30° projection (Figure 3B).

**Figure 4:**
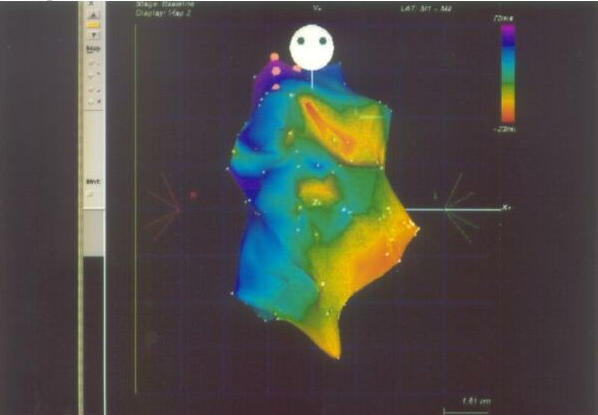
Isochronal mapping on CARTO system of right ventricle showing earliest activation (red color) in the outflow tract region suggestive of right ventricular outflow tract ventricular tachycardia (AP projection).

**Figure 5:**
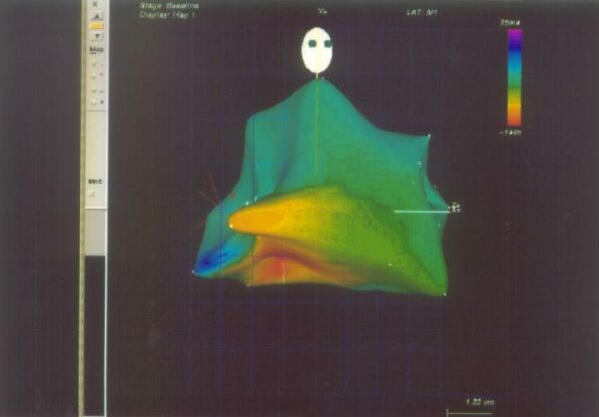
Isochronal mapping on CARTO system of left ventricle showing earliest activation (red color) in the inferobasal region suggestive of left ventricular tachycardia (RAO projection).

The mapping catheter is dragged over the endocardium, sequentially acquiring the location of its tip together with the local electrogram when the catheter is in stable contact with the wall. By sampling the location of the catheter together with the local electrogram from a plurality of endocardial sites, the three-dimensional anatomy of the chamber is reconstructed in real-time. The local activation time is then color-coded and superimposed on the anatomical map with red indicating early-activated sites, blue and purple late activated areas, and yellow and green areas intermediate activation times (Figure 3,4,5).

The stability of the catheter and contact is evaluated at every site by examining the following criteria: 1) local activation time stability, which is defined as the difference in ms between the local activation calculated from two consecutive beats; 2) location stability, defined as the distance in mm between two consecutive gated locations; 3) morphological superpositioning of the intracardaic electrogram recorded on two consecutive beats; and 4) cycle length stability, defined as the difference between the cycle length of the last beat and the median cycle length during the procedure.

The three-dimensional geometry of the chamber is generated using a modified "star" reconstruction algorithm. The sets of points from endocardial surface are used for geometrical reconstruction. It is recommended to map each chamber in a different reconstruction bin ('chamber setup"), when mapping more than one chamber, which can be simultaneously displayed.

The electroanatomical maps can be presented in either two or three dimensions as activation, isochronal, propagation, or voltage maps. The activation maps display the local activation time color-coded overlaid on the reconstructed three-dimensional geometry (Figure 3,4,5). The propagation map shows a dynamic color display of the propagation of the activation wavefront across the reconstructed chamber. The voltage map displays the peak-to-peak amplitude of the electrogram sampled at each site. This value is color-coded, with red and purple indicating areas with the lowest and highest amplitude, respectively (Figure 3,4,5). The abnormal low voltage usually represents scar tissue and thus may help in understanding the mechanism underlying the arrhythmia.

#### Clinical applications

The capabilities of the CARTO system to associate relevant electrophysiological information with the appropriate spatial location in the heart and to accurately determine the three-dimensional location and orientation of the ablation catheter may be of great value in guiding ablation. The technology enables one to perform the mapping procedure and to potentially define the mechanism underlying the arrhythmia, to design an ablation strategy, and finally to return accurately to the desired site for the delivery of RF energy.

The CARTO nonfluoroscopic electroanatomic mapping system enhances our ability to analyze and ablate the arrhythmia in following ways: 1) it allows us to associate the intracardiac electrogram with the reconstructed three-dimension anatomy (electroanatomic mapping), which helps to determine the site of arrhythmia origin, its mechanism, and the importance of particular cardiac structures in sustaining the arrhythmia circuit. Knowledge of the arrhythmia mechanism and circuit (e.g., macroreentrant circuit versus focal origin) is critical in planning arrhythmia ablation. Thus, macroreentrant tachyarrhythmias, such as incisional atrial tachycardia, are best mapped using the CARTO system [2,9]) Catheter can be relocated accurately to the points of interest. This enables RF ablation to be delivered by an anatomic approach. This method is particularly useful in ablation of hemodynamically unstable arrhythmias such as ventricular tachycardia, because information can be acquired and stored from multiple short runs of tachycardia and ablation then performed in sinus rhythm [3,10,11]) important cardiac structures such as the His bundle, ostia of venous structures, valve annuli and scar tissue can be tagged properly, which are avoided during ablation. The ability to tag previously unsuccessful ablation sites also helps to identify the correct location for ablation, which may be useful in difficult cases of arrhythmia such as atrial tachycardia or VT of focal origin or of right free wall accessory pathway. 4) Electroanatomical mapping, although not essential for ablating typical isthmus dependent atrial Flutter, is quite useful in a recurrent atrial flutter in locating breakthrough points in the line of block that has been created earlier. The advantage is greater when dealing with postoperative incisional flutter circuits. It also considerably reduces fluoroscopic exposure to radiation for the operator.

#### Limitations

The system is limited to point-to-point mapping strategy. Thus, if the arrhythmias are non-sustained or quickly change to a different morphology or mechanism, e.g., atrial tachycardia degenerates to atrial fibrillation, mapping of such arrhythmias will be difficult to complete and the procedure is likely to take considerable time.

### 2) Basket Catheter mapping

Simultaneous mapping of multiple points has been performed using 64 -pole endocardial basket catheter (Constellation, EP Technologies), which can be deployed percutaneously. Current designs of basket arrays consist of a series of equally spaced electrode pairs mounted on eight flexible splines (A-H), and each spline contains eight 1.5 mm electrodes with 3-mm spacing, which can be straightened and advance from a percutaneous sheath into the cardiac chamber of interest so that the splines deploy and are apposed against the endocardium (Figure 6A& B.

The basket catheter was connected via the amplifier to the three-dimensional mapping system, which provides three-dimensional color construction of electrical activity. The signals are filtered from 30 to 400 Hz. Bipolar mapping is performed using signals from the electrodes on all eight splines.

Resolution is limited to the proportion of electrodes that are in contact with the endocardium and by unequal deployment and spacing of the splines, especially if the geometry is distorted, for example by left ventricular aneurysm. Nevertheless, successful ablation of ventricular tachycardia in humans guided by basket mapping has been described [12]. Basket catheters have also been deployed to assess right atrial activation patterns around the crista terminalis before and after ablation, although the baskets were withdrawn prior to the ablation procedures [13,14]. A basket catheter has also been used to characterize atrial fibrillation and demonstrate organization of atrial electrograms in basket electrodes prior to atrial fibrillation termination [15]. Although the spatial resolution requires interpolation of data, current design of basket mapping systems are able to display animated activation maps and also have catheter location systems, which allow the operator to guide an ablation catheter to a particular electrode on the basket.

#### Advantages of Basket Catheter:

 1) It provides simultaneous, multiple, stable recordings for most of the endocardial surface. Depending on the location of the arrhythmogenic substrate a complete coverage of the reentry circuit is possible. 2) In addition to multiple simultaneous recordings, the basket catheter provides stable, multiple pacing sites distributed throughout the chamber where it is deployed. Thus, the entrainment of arrhythmias could be remarkably facilitated. 3) The color-coded animation images simplify the analysis of multielectrode recordings and help in establishing the relationship between activation patterns and anatomic structures. 4) In patients with atrial flutter, mapping with a basket catheter provides the unique ability of recording from both the sides of the Tricuspid valve and Inferior vena cava (TV-IVC) isthmus simultaneously. 5) It allows high density mapping from infrequent ectopic beats. 6)By mapping the precise location, the number of RF ablations may be reduced substantially.

#### Limitations

 Basket catheters do not at all points orient themselves towards the endocardium in the shape expected of them and this may vitiate our judgment. Basket catheters have a relatively large area near the shaft without electrodes, therefore during mapping some part of endocardium is not in contact with the basket. The quality of recordings is critically dependent on proper selection of basket size. There is a potential risk of thrombo-embolism with left sided mapping.

### 3) Real-time positional management (Cardiac Pathways) EP system

#### Reference and ablation catheters

**Figure 7:**
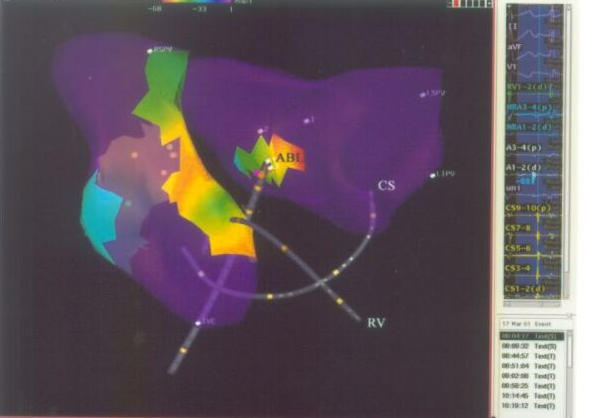
Isochronal mapping on Real Position Management System, showing the reference catheter position in coronary sinus and right atrium; and, ablation / mapping catheter in left atrium (AP projection). CS= Coronary Sinus; RV= Right ventricle; ABL= ablation catheter; RSPV= Right superior pulmonary vein; LSPV= left superior pulmonary vein; LIPV= left inferior pulmonary vein.

Two reference catheters and one mapping/ablation catheter are introduced percutaneously using subclavian and /or femoral approach. One reference catheter is positioned in the coronary sinus, and the other in the right ventricular (RV) apex. For ablation purpose, a 7F, 4-mm tip bidirectional steerable cooled ablation catheter (Cardiac Pathways, Sunnyvale, CA, USA) is used (Figure 7). The cooled-tip ablation catheter can be used in cooled or standard RF non-cooled mode. The reference catheters (Cardiac Pathways) have a 6-F fixed curve distal shaft. The shaft of the coronary sinus reference catheter contains nine 1-mm ring electrodes and one 2-mm tip electrode (interelectrode distance 1 mm), whereas the RV reference and the ablation catheters contain three 1-mm ring electrodes and one 4-mm tip electrodes (interelectrode distance 1 mm). The reference catheters are equipped with four-ultrasound transducers and the ablation catheter contains three ultrasound transducers. The ultrasound transmitter device sends a continuous cycle of ultrasound pulses (558.5 kHz) to the transducers of the reference and ablation catheters. By measuring the time delay from the departure of a transmitted ultrasound pulse and the reception of this pulse at the other transducers, assuming a speed of sound in blood of 1,550 m/sec, the distance between the individual transducers can be calculated. These data are used to define the location of the catheter(s) within the reference frame. Once the 3D-reference frame is established, triangulation can be used to track the position of additional transducers.

Because dimensional and structural characteristics of the catheter are known, it is possible to construct a real-time 3D graphic representation of the catheters, including the position of the electrodes and the transducer. As one of the transducers is positioned distal to the deflection point of the shaft of the catheter, it is possible to display the curvature of the catheter as well. Furthermore, the real-time position management system graphically displays the beat-to-beat movement of the tip of the catheters.

#### Real-time position management (RPM) mapping system

**Figure 8:**
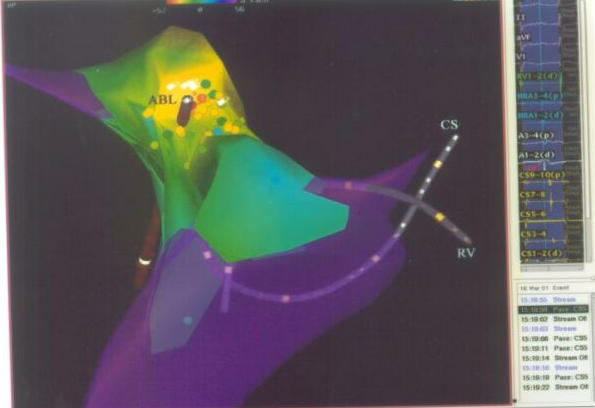
Isochronal map of right atrium on Cardiac Pathways System showing the earliest activation site (red color) at the junction of right atrium and superior vena cava (AP Projection). The right panel of the picture shows earliest activation signal in the ablation catheter (56 ms early from the reference catheter). CS= Coronary Sinus; RV= Right ventricle; ABL= ablation catheter.

The 3D real-time position management and mapping system (Cardiac Pathways) uses ultrasound-ranging techniques to determine the position of a mapping/ ablation catheter relative to two reference catheters. The mapping system consists of an acquisition module and an ultrasound transmitter and receiver unit, both connected to a SPARC 20 computer (Sun Microsystems, Palo Alto, CA, USA). The system is capable of simultaneously processing (1) seven position management catheters, (2) 24 bipolar/48 unipolar electrogram signals, (3) 12- lead ECG and (4) two pressure signals. Signals are sampled at 3kHz per channel with a resolution of 14 bits. The high-pass filters are set at 30 Hz and the low-pass filters at 500 Hz. Electrograms and catheter positions are stored on optical disk. The original position of the reference catheters can be displayed on the real- time window, thereby allowing repositioning of catheters after displacement (Figure 8).

The real-time display of the catheter tip and the possibility of on-line retrieving previous positions and curves of a catheter facilitate repositioning of a catheter at previously marked sites. Use of this system may result in reduced fluoroscopy time. Routine application of the real-time position management system requires only the use of special catheters; no additional catheters or skin electrodes are needed. As any type of catheter containing ultrasound transducers can be "tracked" within the reference frame and used to locate positions, it is possible to position, for example, two catheters simultaneously in the RA and the LA to create lesions using this guidance system. This may be useful in case of the creation of linear lesions in atrial fibrillation patients [16]. The ability to create lesions within a defined area will allow systematic ablation of endocardial zones of slow conduction critical for the perpetuation of reentrant VT [27,18].

The limitation of this system is occasional failure of ultrasound transducers, requiring replacement of a catheter. Secondly, a "Voltage map" cannot be obtained unlike the other systems described.

## Conclusion

RF ablation has become first-line therapy for many tachyarrhythmias. It has a high short-term success rate, low complication rate, and results in improved quality of life compared with other treatments. One concern is the potential effect of radiation exposure to both patient and medical personnel especially during long procedures. The newer mapping system may be helpful in this regard. Mapping systems does reduce fluoroscopic time for ablation without compromising efficacy or safety. The procedure time however may be even longer with the newer mapping systems.

These newer mapping systems are useful tool for guiding catheter ablation of unstable arrhythmias (e.g., ventricular tachycardia) and complex atrial arrhythmias. The newer mapping systems at present are not cost-effective for developing countries. Since the special catheters used for nonfluoroscopic mapping are relatively expensive than the conventional catheters, they should not be used with due consideration, especially for arrhythmias that are relatively simple and easy to ablate, such as AVNRT, preexcitation syndrome, or AV nodal junctional ablation.

Furthermore, these systems are continually being refined and the maps obtained from these systems are not ideal yet. The learning curve with the operators with this new and rapidly advancing technology is also an issue. Currently, these systems remain an adjunct to the conventional methods of mapping to guide catheter ablation. More research and study is required to define the primary indications for their usage.

## Figures and Tables

**Figure 6A:**
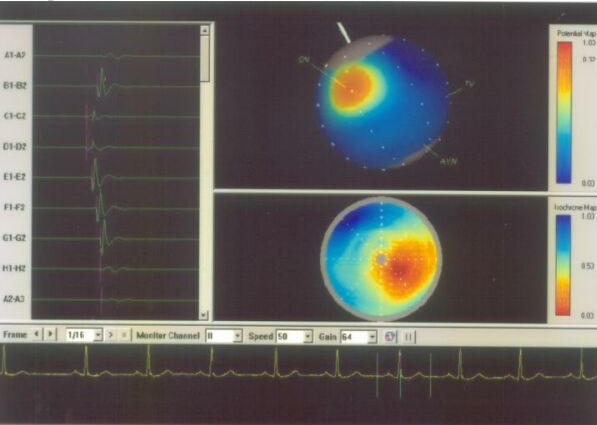
Basket mapping of normal sinus rhythm. The right upper panel shows the potential map, the earliest activation (red color) at the site of sinus node. In addition, the splines of basket catheters are aligned with the endocardial wall. The right lower panel shows isochronal map of the sinus rhythm. The left panel shows the electrogram from the different splines. SN= Sinus node; TV= tricuspid valve; AVN= Atrioventricular node.

**Figure 6B:**
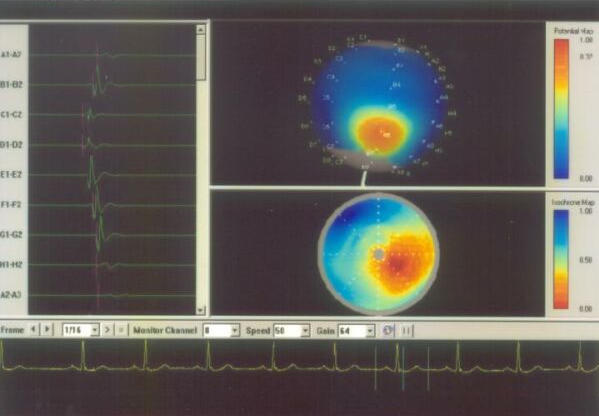
Shows different splines of basket catheter in the right atrium. The isochronal and potential maps are suggestive of ectopic atrial beat.
